# Mortality in patients with Sanfilippo syndrome

**DOI:** 10.1186/s13023-017-0717-y

**Published:** 2017-10-23

**Authors:** Christine Lavery, Chris J. Hendriksz, Simon A. Jones

**Affiliations:** 1 Society of Mucopolysaccharide Diseases, MPS House, Repton Place, White Lion Road, Amersham, HP7 9LP UK; 2 Adult Inherited Metabolic Disorders, The Mark Holland Metabolic Unit, Salford, UK; 3 Manchester Centre for Genomic Medicine, Manchester, UK

**Keywords:** Sanfilippo syndrome, Mucopolysaccharidosis, Mortality, Respiratory failure, Pneumonia

## Abstract

**Background:**

Sanfilippo syndrome (mucopolysaccharidosis type III; MPS III) is an inherited monogenic lysosomal storage disorder divided into subtypes A, B, C and D. Each subtype is characterized by deficiency of a different enzyme participating in metabolism of heparan sulphate. The resultant accumulation of this substrate in bodily tissues causes various malfunctions of organs, ultimately leading to premature death. Eighty-four, 24 and 5 death certificates of patients with Sanfilippo syndrome types A, B and C, respectively, were obtained from the Society of Mucopolysaccharide Diseases (UK) to better understand the natural course of these conditions, covering the years 1977–2007.

**Results:**

In Sanfilippo syndrome type A mean age at death (± standard deviation) was 15.22 ± 4.22 years, 18.91 ± 7.33 years for patients with Sanfilippo syndrome type B and 23.43 ± 9.47 years in Sanfilippo syndrome type C. Patients with Sanfilippo syndrome type A showed significant increase in longevity over the period of observation (*p* = 0.012). Survival rates of patients with Sanfilippo syndrome type B did not show a statistically significant improvement (*p* = 0.134). In Sanfilippo syndrome types A and B, pneumonia was identified as the leading cause of death.

**Conclusions:**

The analysis of 113 death certificates of patients with Sanfilippo syndrome in the UK has demonstrated that the longevity has improved significantly in patients with Sanfilippo syndrome type A over a last few decades. The numbers of patients with Sanfilippo syndrome types B and C were too small to identify any significant trend changes for these groups. Respiratory tract infections, notably pneumonia, remain the leading cause of mortality in Sanfilippo syndrome types A and B. The extended lifespans of patients with Sanfilippo syndrome type A were achieved despite the lack of therapies to target the primary insult or pathophysiology of the disease. However, the mean age at death of these patients remains low when compared with the general population. Therefore, there is an urgent need for effective disease-specific therapies to be developed so that the quality of life and survival of patients with Sanfilippo syndrome can be improved.

## Background

Sanfilippo syndrome (mucopolysaccharidosis type III) belongs to the group of approximately 50 inherited monogenic lysosomal storage disorders [1]. Currently, there are four autosomal recessive subtypes of Sanfilippo syndrome (A, B, C and D) recognized in humans [2]. Each of them results from deficiency of a different enzyme responsible for the degradation of a common glycosaminoglycan (GAG), heparan sulphate, a macromolecule normally localized in the extracellular matrix. Sanfilippo syndrome type A, B, C and D result from deficiency of heparan-*N*-sulphatase [3], *N*-acetyl-α-glucosaminidase [4], α-glucosaminidase *N*-acetyltransferase [5] and *N*-acetylglucosamine 6-sulphatase [6], respectively.

The incidence of Sanfilippo syndrome varies according to subtype and geographical region [7]. Sanfilippo syndrome type A is more common in northern Europe than in Mediterranean countries (incidence in The Netherlands 1.16 per 100,000 live births; Greece 0.00 per 100,000 live births) [8, 9]. By contrast, Sanfilippo syndrome type B is more prevalent in southern Europe than in the north (eg incidence in Greece and Sweden is 0.78 and 0.03 per 100,000 live births, respectively) [8, 10]. Sanfilippo syndrome type C is less common than subtypes A and B, with an incidence of 0.06 per 100,000 live births in the United Kingdom and 0.21 per 100,000 live births in The Netherlands [9, 11]. The least common subtype of Sanfilippo syndrome is type D, with an incidence of 1 per 1,000,000 live births [2, 12].

The deficient breakdown and subsequent build-up of heparan sulphate in bodily tissues causes a variety of clinical anomalies, mainly presenting in the nervous system but also resulting in musculoskeletal, respiratory, ear, nose and throat, gastroenterological, cardiac and ocular manifestations [13, 14]. Overall, the somatic changes in patients with Sanfilippo syndrome, such as coarseness of facial features, broad eyebrows, dark eyelashes, dry and rough hair, hearing loss, hepatosplenomegaly, skeletal pathology that affects growth and causes degenerative joint disease, are less pronounced than in patients suffering from other forms of mucopolysaccharidosis, such as Hurler or Hunter syndromes [2, 7, 15–18].

The natural history of Sanfilippo syndrome can be divided into three phases [7]. The first stage begins when a child starts exhibiting mild developmental delay accompanied by somatic manifestations, such as recurrent ear, nose and throat disease or bowel disturbance, normally between ages 1 and 4 years [7, 8, 17–20]. The subsequent phase is characterized by the appearance of behavioural difficulties, such as a combination of hyperactivity and sleep disturbances, when a child is 3–5 years old [17, 21–23]. Patients may remain in this state for 5–10 years, after which there is a progressive and severe loss of intellectual processes and motor functions [6]. However, a more rapidly progressing phenotype that manifested with cognitive loss at 42–48 months has also been reported [24]; demonstrating that Sanfilippo syndrome type A shows heterogeneity in the onset and rate of cognitive decline [24]. In general, it has been observed that patients suffering from Sanfilippo syndrome lose the ability to speak before they cease to walk [15, 25]. Ultimately, individuals with this condition regress to a vegetative state that lasts until death [17, 18, 26–29] and seizures are common in this final stage [7, 30].

There is currently no cure or standard treatment for patients with Sanfilippo syndrome [6, 7]. In the absence of effective therapies, patient care is limited to symptom management and palliative support [6]. Disease-specific treatments for Sanfilippo syndrome are being studied, including forms of enzyme replacement therapy (ERT), substrate reduction therapy, bone marrow transplantation and gene therapy, with some reaching the mid-to-late stages of clinical development [6, 31]. To be able to optimize current management and evaluate the effectiveness of novel treatments for Sanfilippo syndrome, it is important to understand the natural progression of the disease, life-expectancy and common causes of death.

Here, we analyze survival and causes of death of patients with Sanfilippo syndrome and how these have changed in recent decades, using data collected by the Society for Mucopolysaccharide Diseases (UK). These data will be of interest to clinicians, healthcare authorities and commissioning bodies, as well as to patients, their families and patient societies.

## Methods

The Society for Mucopolysaccharide Diseases (UK) made available death certificates of all deceased patients with Sanfilippo syndrome types A, B and C held in its database. The death certificates provided information on date of birth, gender, date of death and primary cause of death. The Society has aimed to collect data on every patient with Sanfilippo syndrome type A, B and C in the UK. The number of patients missing from the database is unknown; however, it is estimated by the collators to be very few because most, if not all, individuals with Sanfilippo syndrome type A, B and C in the UK are treated at a small number of designated centres.

Microsoft® Excel® was used to perform the statistical analysis. The Student’s two-tailed *t*-test was applied to determine statistical significance.

## Results

### Patient characteristics

Death certificates were available for 84 patients (42 male, 42 female) with Sanfilippo syndrome type A, covering the years 1977–2007, and for 24 patients (14 male, 10 female) with Sanfilippo syndrome type B, covering years 1983–2007. No death certificates were available for patients with Sanfilippo syndrome type B before 1983. Death certificates were available for only five patients (three male, two female) with Sanfilippo syndrome C, covering years 1989–2003 (Table 1). No death certificates were available for patients with Sanfilippo syndrome type C before 1989. Owing to the small number of death certificates available, no further analysis of patients with Sanfilippo syndrome type C is included in this article. Death certificates were not available for Sanfilippo syndrome type D, and thus this disorder has not been discussed further in this study.

**Table 1:** Patient characteristics

	Disease subtype		
	A	B	C
Number of patients	84	24	5
Male	42	14	3
Female	42	10	2
Mean age at death, years	15.22	18.91	23.43
Median age at death, years	14.50	15.42	22.42
Standard deviation, years	4.22	7.33	9.47

### Changes in longevity over time

**Fig. 1:**
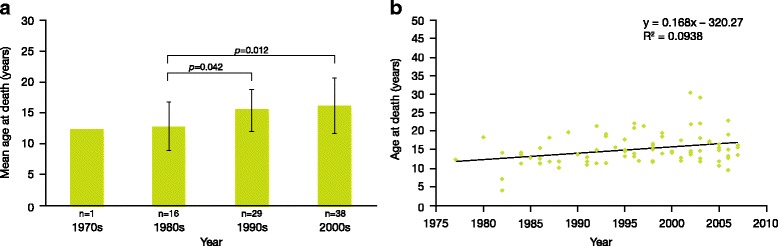
Longevity in patients with Sanfilippo syndrome A. **a** Mean age at death over time of patients with Sanfilippo syndrome type A (n indicates the number of patients); **b** age at death by individual patient with Sanfilippo syndrome type A

Mean age at death of patients with Sanfilippo syndrome type A was 15.22 ± 4.22 years (mean age at death ± standard deviation [SD]). Moreover, the mean age at death of the patients with Sanfilippo syndrome type A showed significant improvement between the 1980s and 1990s (*p* = 0.042) and between the 1980s and 2000s (*p* = 0.012) (Fig. 1a). A statistical assessment of the increase in longevity could not be performed for 1970s because only one patient died during that period. An analysis of longevity in patients with Sanfilippo syndrome type A showed a weak trend over time towards gradual improvement in life-expectancy (*R* ^*2*^ = 0.0938; Fig. 1b).

**Fig. 2:**
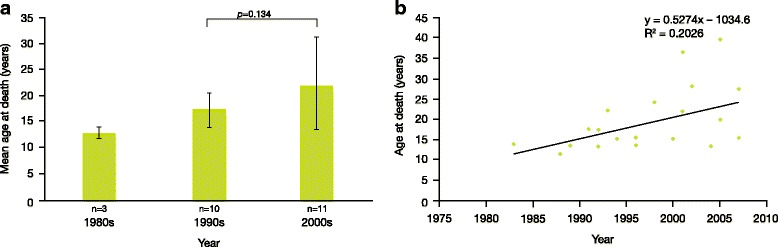
Longevity in patients with Sanfilippo syndrome B. **a** Mean age at death over time of patients with Sanfilippo syndrome type B (n indicates the number of patients); **b** age at death by individual patient with Sanfilippo syndrome type B

Mean age at death (± SD) of patients with Sanfilippo syndrome type B was 18.91 ± 7.33 years (Table 1). An analysis of longevity in patients with Sanfilippo syndrome type B showed a trend over time towards steady improvement in life-expectancy (*R* ^*2*^ = 0.2026; Fig. 2b). However, this group did not show a statistically significant improvement in longevity between the 1990s and 2000s (*p* = 0.134) (Fig. 2a). A statistical assessment was not conducted for 1980s because only three patients died during this period, thus raising questions about statistical power of such analysis. Mean age at death (± SD) of patients with Sanfilippo syndrome type C was 23.43 ± 9.47 years (Table 1).

The mean age at death for patients with Sanfilippo syndrome type A was lower (15.22 ± 4.22 years) than for patients with Sanfilippo syndrome type B (18.91 ± 7.33 years). This difference was statistically significant (*p* = 0.029). The mean age at death of patients with Sanfilippo syndrome type C was higher than for both A and B subtypes (23.43 ± 9.47 years). However, because of the small size of Sanfilippo syndrome type C cohort, the statistical significance of this result could not be tested.

### Primary cause of death

**Fig. 3:**
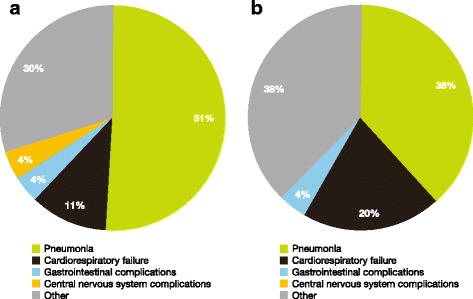
Primary cause of death in patients with Sanfilippo syndrome types A and B. **a** Primary cause of death in patients with Sanfilippo syndrome A; **b** primary cause of death in patients with Sanfilippo syndrome B (there were no mortalities caused by central nervous system complications in this group)

Pneumonia accounted for more than 50% of deaths in patients with Sanfilippo syndrome type A (Fig. 3a). Cardiorespiratory failure, gastrointestinal complications, central nervous system complications and other causes were responsible for 11%, 4%, 4% and 30% of deaths, respectively (Fig. 3a).

Pneumonia was also the leading cause of death in patients with Sanfilippo syndrome type B (38%; Fig. 3b). Cardiorespiratory failure, gastrointestinal complications and other causes accounted for 20%, 4% and 38% of deaths, respectively (Fig. 3b).

Primary causes of death in patients with Sanfilippo syndrome type A that were not categorized as pneumonia, cardiorespiratory, gastrointestinal and central nervous system-related, included: accidental suffocation, sudden unexpected death syndrome, renal failure and Sanfilippo syndrome type A only. One patient was misclassified as having died of Duchenne muscular dystrophy. Primary causes of death in patients with Sanfilippo syndrome type B that were not categorized as pneumonia, cardiorespiratory and gastrointestinal included: multi-organ failure, sepsis and Sanfilippo syndrome type B only. Death certificates of patients for whom the primary cause of death was indicated as Sanfilippo syndrome type A or B did not contain any additional information about which possible complication resulted in patient’s demise.

### Differences in primary cause of death by decade

There was a decrease in the proportion of deaths caused by pneumonia from 1980s to 2000s in patients with Sanfilippo syndrome types A. The proportion of deaths caused by pneumonia was 60%, 59% and 45% for 1980s, 1990s and 2000s, respectively. There was only one patient with Sanfilippo syndrome type A in 1970s and he died from central nervous system complications. The proportion of deaths caused by pneumonia also showed a general decline from 1980s to 2000s in patients with Sanfilippo syndrome type B. The proportion of deaths caused by pneumonia was 67%, 30% and 36% for 1980s, 1990s and 2000s, respectively. Although the mean age at death due to pneumonia increased over time in patients suffering from Sanfilippo syndrome type A from 13.21 years in the 1980s to 16.63 years in the 2000s, this increase was not statistically significant (*p* = 0.070). The statistical analysis of pneumonia in Sanfilippo syndrome type B was not possible due to a small cohort size.

## Discussion

Mean and median ages at death of patients with Sanfilippo syndrome types A, B and C in the UK were below 25 years, which was in line with previous studies that examined the natural history of patients with Sanfilippo syndrome. A study of 22 Swedish children with Sanfilippo syndrome type A reported that median age at death was 16.2 years for these patients [25], whereas a retrospective review of 46 children with Sanfilippo syndrome type A conducted by researchers of Children’s Hospital of Pittsburgh, USA, demonstrated that Kaplan − Meier analysis showed a 60% probability of children surviving beyond 17 years of age [15]. Another study on Sanfilippo syndrome types A, B and C conducted in Spain, showed that the median age at death of children with Sanfilippo syndrome A was 15 years. The median age at death for one patient with Sanfilippo syndrome type B was 19 years in the same investigation [32].

In contrast to the above reports, a study conducted in The Netherlands showed that the median age at death of 18 patients with Sanfilippo syndrome type B was 43.5 years (range 13–69). The longer-than-anticipated longevity of these patients was attributed to attenuated phenotypic manifestations [28]. Another study of Dutch patients with Sanfilippo syndrome type B showed that six individuals died between the ages of 28 and 69 years [33]. On the other hand, our study indicated that median age at death of male (15.42 years) and female (20.83 years) patients with Sanfilippo syndrome type B was lower than in both the Dutch studies, with the range of 11.33–39.67 years for all patients. This discrepancy might reflect a predominance towards a more severe phenotype of our comparatively large cohort.

Patients with Sanfilippo syndrome type A showed a statistically significant improvement in life-expectancy over time. Although the longevity of patients with Sanfilippo syndrome type B also seemed to increase, it was not statistically significant. However, the mean age at death for patients with Sanfilippo syndrome type B was higher than for the Sanfilippo syndrome type A cohort. This result was supported by the statistical analysis. The mean and median ages at death of patients with Sanfilippo syndrome type C were higher than for individuals with either Sanfilippo syndrome type A or B. However, due to a small size of the Sanfilippo syndrome type C cohort, this conclusion could not be tested by statistical analysis. The study conducted in The Netherlands showed that the patients with Sanfilippo syndrome type C had a milder course than previously reported and the mean age at death of the 12 patients was 34 years [17]. Although, the mean age at death of patients with Sanfilippo syndrome type C in our study was lower (ie 23.43 years), there were patients who reached the same age as individuals from the Dutch study (range 9.67–34.25 years). The difference in the outcomes of these two studies may be ascribed to the small size of our cohort (five patients only).

Pneumonia remains the primary cause of death in patients with Sanfilippo syndrome types A and B. The study of Spanish patients with Sanfilippo syndrome types A and B showed that the cause of death in six patients was respiratory infection, whereas four subjects died due to cardiorespiratory failure [32]. The Dutch researchers describing the natural history of patients with Sanfilippo syndrome type B, also identified pneumonia (*n* = 7) as a leading cause of death [28], which is in line with our results. The same investigation showed that the other causes of death included withdrawal of artificial feeding because of profound functional disabilities (*n* = 2), accidental strangulation in restraints (*n* = 1), pancreatitis (*n* = 1), surgical complications (*n* = 1), aspiration of blood during a severe bleeding (n = 1), heart failure (*n* = 1) and unknown cause (*n* = 3). Another Dutch study [33], which examined natural history of patients with Sanfilippo syndrome type B, discovered that the main causes of death were pneumonia, sepsis or cachexia after recurrent aspiration pneumonias. Moreover, it was indicated in this study that nearly all subjects were prone to infections. Eleven patients suffered from pneumonia at least once, nine patients had recurrent otitis and 10 had at least one episode of cystitis. Finally, the Dutch investigation on patients with Sanfilippo syndrome type C showed again that the leading cause of death was respiratory infection [17]. Therefore, the results of our analysis were in line with the investigations performed in Europe and the USA.

Most mortalities described in this analysis were categorized as pneumonia. Similar findings have been reported for other progressive diseases; two studies have shown that respiratory complications are observed more frequently in patients with neurological impairment and result in more hospitalizations compared with patients without neuromuscular or neurodevelopmental disorders [34, 35]. This suggests that the trend towards pneumonia and cardiorespiratory failure observed in this study is likely to be secondary to the central nervous system manifestations of the disease rather than a direct result of GAG accumulation. Despite the number of mortalities categorized as pneumonia, our study showed that the proportion of deaths caused by this complication has decreased in recent decades in patients with Sanfilippo syndrome types A and B. However, it should be noted that despite the lower proportion of pneumonia among patients with Sanfilippo syndrome, the longevity of patients dying from this respiratory infection has not increased significantly. It is possible that efforts to improve disease management and multidisciplinary care and to refer patients to specialist centres are being reflected in lower number of patients with Sanfilippo syndrome types A and B dying due to pneumonia. Common use of gastrostomy feeding has reduced the risk of aspiration pneumonia and may have also contributed to the lower death rate related to pulmonary disease [25]. As was suggested previously in patients with Morquio syndrome type A [36], improvements in pulmonary care, regular immunizations, prompt and aggressive treatment of respiratory tract infections, prevention of weight gain and availability of respiratory support may have also contributed to the lower proportion of pneumonia deaths among patients with Sanfilippo syndrome types A and B.

It is unclear from our report whether instances of cardiorespiratory failure were driven by respiratory failure or whether they were of pure cardiac origin. In the other MPS disorders cardiac disease is becoming an increasing problem in older patients and this trend may be observed in patients with Sanfilippo syndrome in the future [13, 37, 38].

There is currently no cure for Sanfilippo syndrome as ERT does not cross the blood − brain barrier and bone marrow transplantation does not appear to produce sufficient levels of missing enzymes to prevent accumulation of GAGs in the bodily tissues and neurological deterioration of patients. Existing approaches to patient management are directed towards ameliorating sleep disturbances and regulating behavioural abnormalities. There are several promising therapies under development for Sanfilippo syndrome. They include injection of the relevant recombinant enzyme into the cerebrospinal fluid, gene therapy and substrate reduction therapy. However, the safety and effectiveness of these approaches is still being studied and their impact on mortality in patients with Sanfilippo syndrome will not be known for many years [6, 31].

A limitation of this study is the use of death certificates to determine primary causes of death in patients with Sanfilippo syndrome. In many instances, death certificates listed not only primary causes of mortality but also secondary and tertiary causes that were occasionally conflicting. Also, in many cases, the information provided by death certificates was limited to a few words (eg Sanfilippo syndrome), thus restricting precise determination of how patients died. Another concern when using death certificates to identify primary causes of demise is a lack of knowledge regarding Sanfilippo syndrome. This may result in an incorrect interpretation of a real cause of death (eg cardiorespiratory failure instead of neurological complications). Listing a cardiorespiratory failure as a primary cause of death may also be a limiting factor. In patients with Sanfilippo syndrome, cardiorespiratory failure may be an extension of the underlying neurological disease to brain stem/medulla, which controls respiration and cardiac functions. Therefore, ascribing a primary cause of death to cardiorespiratory reasons may disguise a real cause of mortality. Consequently, it is important to consider problems with accuracy when using the death certificates. On the other hand, these certificates provide valuable information about patient longevity that gives vital clues as to how improvements in patient care have impacted clinical outcomes over recent decades.

Another limitation of this study is the absence of clinical notes for the patients. It is not known what the course of disease has been for individual patients. Furthermore, there is no information available about each patient’s functional ability and the presence or absence of comorbidities, which may have provided useful context for the trends observed in this study.

## Conclusion

The findings of this study suggest that survival of patients with Sanfilippo syndrome type A in the UK has improved in recent decades despite the lack of therapies that could target the underlying pathophysiology of the disease. The primary cause of death in these patients is pneumonia, although the proportion of patients dying from this respiratory infection has decreased, perhaps reflecting improvements in respiratory care.
